# Simultaneous Esophageal and Gastric Ulceration Due to Doxycycline Ingestion: Case Report and Review of the Literature

**DOI:** 10.4021/gr498w

**Published:** 2012-11-20

**Authors:** Aviva Leber, Jeff Stal

**Affiliations:** a Division of Gastroenterology, Department of Medicine, Women’s College Hospital , University of Toronto, Canada

**Keywords:** Esophageal ulcer, Gastric ulcer, Doxycycline

## Abstract

Doxycycline induced mucosal injury to the esophagus and, rarely, stomach is an under appreciated cause of odonyphagia and chest pain. The diagnosis is dependent on a comprehensive exposure history and endoscopic evaluation. Once recognized, doxycycline induced ulceration is easily treated with cessation of the medication and gastric acid suppression. Our case report describes a classic description of doxycyline induced esophageal ulceration with the unusual endoscopic finding of a simultaneous antral ulcer.

## Introduction

Esophageal and gastric mucosa is regularly exposed to ingested substances that have the potential to cause mucosal damage and ulceration. Gastric ulceration from non-steroidal anti-inflammatory medications is the best-known example. Doxycycline is a less well-recognized agent that can frequently cause esophageal ulceration and rarely gastric ulcers. The diagnosis is dependent on a comprehensive medical history, including a complete exposure history and endoscopic evaluation. Once recognized, symptoms and endoscopic findings resolve quickly by cessation of offending agent and gastric acid suppression therapy. This case report describes the uncommon presentation of simultaneous esophageal and gastric ulceration in a patient using doxycycline for acne.

## Case Report

A 28-year-old woman presented to an emergency room with a three day history of a new retrosternal chest pain and odynophagia. The pain had a burning quality and was located in her restrosternally. It was constant in nature with fluctuations in severity. Swallowing solids and liquids significantly aggravated her pain. There was no history of dysphagia, abdominal pain, fever, melena, rectal bleeding or weight loss. She had never experienced these symptoms before.

Her past medical history was significant only for acne, which had been treated medically with doxycycline for the past six months. She was a non-smoker and consumed only minimal amounts of alcohol. There was no recent travel history or infectious contacts. Her physical examination was unremarkable with no evidence of oral thrush.

Initial investigations in the emergency room revealed a normal complete blood count and a negative troponin and B-HCG. Her CXR was normal. She was discharged home after several hours and given a prescription for rabeprazole 20 mg bid. She remained on the doxycycline. She was referred to a gastroenterologist and a gastroscopy was arranged to investigate the etiology of her symptoms.

**Figure 1:**
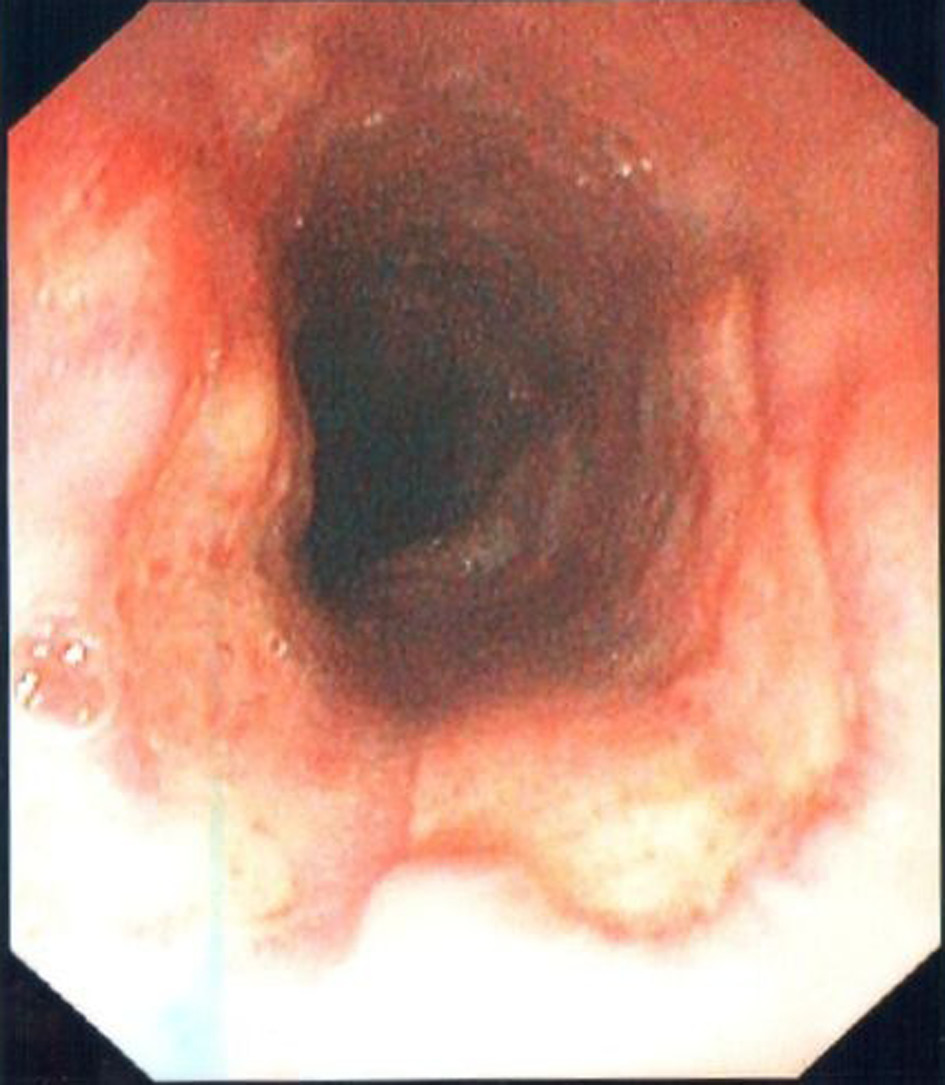
Large semi-circumferential mid esophageal ulcer.

**Figure 2:**
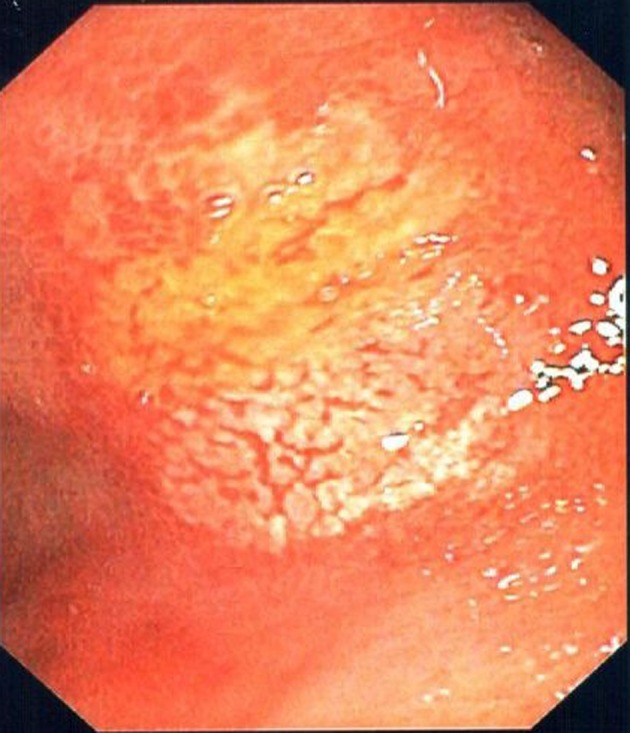
Gastric antral ulcer.

The gastroscopy revealed a large almost circumferential ulcer in the mid-esophagus as well as a peculiar-looking area of white plaque with an erythematous base in the proximal antrum (Fig. 1, 2). Brushings and biopsies were taken for candida, HSV, CMV, AFB, H. pylori and routine histology. She was advised to discontinue the doxycycline following completion of the gastroscopy. She remained on rabeprazole. All microbiologic investigations from the gastroscopy biopsies were negative.

Histologically the esophageal biopsies demonstrated underlying squamous epithelium with acute inflammation and ulceration. The gastric biopsies were significant for a neutrophilic infiltration with diffuse erosion and a fibropurulent exudate on the surface. Although the histology was not specific, it was consistent with a drug-induced injury.

Her symptoms improved quickly and dramatically upon cessation of doxycycline. A repeat gastroscopy two months later was normal with complete healing of the esophageal ulcer and gastric lesion. She did not experience any recurrent symptoms and remained off the doxycycline.

## Discussion

Drug induced esophageal ulcers were first reported in the 1970s [1]. The exact incidence of esophageal ulceration due to oral medications is not known but it is estimated that 10,000 cases per year occur in the US [2]. This number is likely an underestimation, as many cases are not recognized since symptoms usually resolve rapidly after cessation of the medication. Antibiotics account for 50-60% of drug related esophageal toxicity [2, 3] and tetracyclines, in particular doxycycline, are commonly implicated. In contrast, very little is known about the occurrence of non-NSAID related gastric ulcerations. The most frequently described culprit medication is potassium chloride. There are only two previous case reports of doxycycline induced gastric ulceration both occurring simultaneously with esophageal ulceration [4, 5].

Doxycycline is believed to act through a direct caustic effect on the esophageal and gastric mucosa, likely due to its acidic nature [5, 6]. In animal studies, direct exposure of esophageal mucosa to tetracycline causes deep ulcerations [6]. Furthermore the pill formulation is an important contributing factor, with a higher risk of esophageal ulcerations associated with capsules due to their gelatinous shell and tendency to stick and lodge in the esophageal mucosa [7]. Patient characteristics that increase the risk of drug induced esophageal and gastric ulcers include known esophageal motility disorders, hiatal hernia, small volume of fluid ingestion with medication and a supine position after taking medication [2]. Age does not seem to be a discriminating factor, and interestingly the previously reported doxycycline-induced gastric ulcerations both occurred in young women.

Clinically, patients usually present due to the esophageal ulcer with a combination of odonyphagia, retrosternal chest pain and dysphagia. In one case series that examined 36 patients with doxycycline related esophageal toxicity, 94% of patients had odonyphagia, 80% had retrosternal chest pain and 54% had dysphagia [3]. Symptoms generally manifest quickly within hours or days of beginning the medication [2]. Gastroscopy is the diagnostic modality of choice. Esophageal injury frequently results in a discrete mid-esophageal ulcer. In the same case series 24/36 ulcers were localized to the mid esophagus and the median number of ulcers per patient was two [3]. The predilection for the mid-esophagus is hypothesized to be secondary to mechanical factors such as the compressive effect of the aortic arch or adjacent left atrium. Gastric ulceration is much less common and findings may be variable. Both previous descriptions involved superficial ulcerations in the cardia and fundus. Histologically, both gastric and esophageal biopsies reveal acute inflammation with loss of epithelial surface, fibrin deposition and polymorphonuclear leukocyte infiltration [4]. Treatment consists of stopping the offending medication and gastric acid suppression. Symptoms generally resolve within one week and one case series of 9 patients from Singapore demonstrated complete healing of the esophagus during repeat endoscopy 2 - 4 weeks later [1]. Significant complications such as perforation or the development of late strictures are rare. Our patient had a classic exposure history and clinical course, but had the unusual endoscopic findings of both esophageal and gastric ulceration.

Our case report describes the rare occurrence of simultaneous esophageal and gastric ulcers due to ingestion of doxycycline and represents the first case report describing a gastric ulcer localized in the antrum. A high index of suspicion and a complete exposure history is necessary to expediently diagnosis and appropriately manage pill induced mucosal injury to the upper GI tract.
